# Association between weight-adjusted waist circumference index and myocardial infarction: results from the national health and nutrition examination survey, 1999–2014

**DOI:** 10.3389/fcvm.2024.1451987

**Published:** 2024-11-18

**Authors:** Jian-hong Yu, Ming-gang Yin

**Affiliations:** Department of Clinical Laboratory, Zigong First People’s Hospital, Zigong, China

**Keywords:** weight-adjusted waist circumference index, myocardial infarction, obesity, National Health and Nutrition Examination Survey (NHANES), cross-sectional study

## Abstract

**Background:**

Weight-adjusted waist circumference index (WWI) is a novel index related to obesity and has been associated with the risk and prognosis of several diseases. The aim of the study was to determine the association between WWI and myocardial infarction.

**Methods:**

The study analyzed cross-sectional data from 31,535 participants derived from the 1999–2014 National Health and Nutrition Examination Survey (NHANES) dataset. Multiple logistic regression and restricted cubic spline (RCS) analyses were conducted to assess both linear and nonlinear associations between WWI and myocardial infarction. Subgroup analyses and interaction tests were also performed.

**Results:**

Among the 31,535 participants analyzed, 1,449 (4.82%) had experienced a myocardial infarction. The fully adjusted models demonstrated a positive association between WWI and myocardial infarction [odds ratio (OR) = 1.34, 95% confidence interval (CI): 1.13–1.58]. Individuals in the highest quartile of WWI were 81% more likely to suffer from a myocardial infarction compared to those in the lowest quartile (OR = 1.81, 95% CI: 1.24–2.63). The analysis employing restricted cubic spline modeling indicated a nonlinear positive correlation between WWI and myocardial infarction. The risk of myocardial infarction was 1.29 times higher when WWI was below 10.97 cm/√kg (OR = 2.29, 95% CI: 1.37–3.84). When WWI exceeded 10.97 cm/√kg, the upward trend in the risk of myocardial infarction significantly slowed with increasing WWI (OR = 1.26, 95%CI:1.03–1.56). A threshold WWI value of greater than 11.15 cm/√kg was identified for predicting myocardial infarction, outperforming waist circumference and body mass index (BMI). Subgroup analyses revealed that the impact of WWI on myocardial infarction varied across different populations. Interaction analyses demonstrated significant interactions between myocardial infarction incidence and WWI with age, hypertension, coronary heart disease, angina pectoris, and stroke (*P* for interaction <0.05), but not with gender, race, obesity, diabetes, or prediabetes and heart failure (*P* for interaction >0.05).

**Conclusions:**

The findings suggest that there is a positive and non-linear association between WWI and the incidence of myocardial infarction. We recommend incorporating WWI into routine physical examinations and cardiovascular risk screening as an early warning mechanism. This may facilitate early identification of high-risk individuals and promote earlier preventive interventions.

## Introduction

Myocardial infarction, a serious cardiovascular condition, occurs due to ischemic and hypoxic necrosis in the myocardium caused by the narrowing or blockage of the coronary arteries (1). Globally, more than 7 million people experience an acute myocardial infarction (AMI) every year, with AMI accounting for around 1.8 million deaths annually (2). Around 85% of all deaths from cardiovascular disease (CVD) are attributed to acute myocardial infarction (AMI) (3). Acute myocardial infarction is increasingly prevalent at a younger age (4–6). Myocardial infarction is characterized by mild initial symptoms, sudden onset, and high mortality, posing challenges for its prevention, diagnosis, and treatment (7). Early recognition of myocardial infarction risk is crucial for disease prevention. The prevalence of obesity and overweight is rapidly increasing in both developed and developing countries, posing serious public health challenges today (8). References estimate that by 2030, 50% of the adult population will be classified as obese (9). Obesity is a risk factor for several diseases, including cardiovascular disease (10). Central obesity indicators, such as waist circumference and waist-to-hip ratio, have a stronger correlation with the risk of cardiovascular disease compared to traditional BMI indicators (11, 12). Higher levels of waist circumference are associated with an increased risk of myocardial infarction (13). Although waist circumference does not adequately capture the effects of central obesity, there is an “obesity paradox”. The weight-adjusted waist index (WWI), an emerging indicator of obesity, combines the advantages of waist measurement with the limitations associated with BMI, providing new perspectives for cardiovascular disease research (14).

Existing literature has demonstrated that the weight-adjusted waist circumference index (WWI) exhibits a positive correlation with the percentage of body fat and a negative correlation with muscle mass (15, 16). Several investigations have identified a significant association between WWI and the risk of developing and prognosticating outcomes of various diseases. Specifically, WWI has been linked to an elevated risk of diabetic nephropathy and cardiovascular disease among diabetic patients (17, 18). Furthermore, in the general population, increased WWI levels have been associated with a heightened risk of kidney stones (19) and gallstones (20). Notably, WWI has also been correlated with an increased likelihood of abdominal aortic calcification (21). A prospective study involving older adults aged 60 and above revealed that elevated WWI was significantly associated with an increased risk of all-cause mortality (22).

Despite the existing studies examining the relationship between WWI and numerous health outcomes, the specific association between WWI levels and the risk of myocardial infarction remains inadequately explored. This study hypothesizes that WWI is positively associated with myocardial infarction risk, with this relationship influenced by demographic characteristics and comorbidities.

### Patients and methods

#### Participants

The study participants were selected from the 1999 to 2014 National Health and Nutrition Examination Survey (NHANES). NHANES is a continuous, multistage, probability sample, cross-sectional, population-based survey that evaluates the health and well-being of the U.S. civilian non-institutionalized population (23). The data used in this study were obtained from the National Center for Health Statistics (NCHS) website, which is part of the Centers for Disease Control and Prevention (CDC), on March 9, 2023.

**Figure 1:**
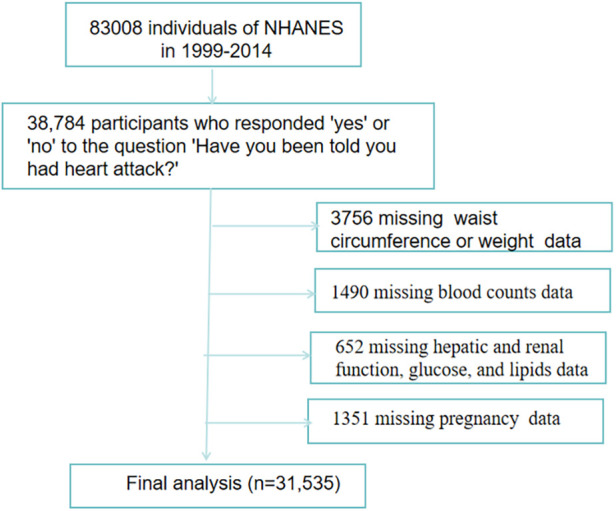
Flow chart of study participants.

The study included 38,784 participants who responded “yes” or “no” to the question “Have you been told you had a heart attack?” on the NHANES questionnaire. Participants were excluded based on the following criteria: (1) missing values on waist circumference or weight (*n* = 3,756); (2) missing values on blood counts (*n* = 1,490); (3) missing values on hepatic and renal function, glucose, and lipids (*n* = 652); and (4) pregnancy (*n* = 1,351). Ultimately, 31,535 individuals were included in the analysis (Figure 1).

### Data collection and measurement

Data collection involved conducting home interviews with participants and laboratory examinations at the Mobile Examination Center (MEC). Trained enumerators used a computer-assisted interview system to collect demographic data. Family members provided the necessary information for participants who were unable to respond. Smoking status was categorized as “never smoking” (less than 100 cigarettes in a lifetime), “former smoking” (smoked more than 100 cigarettes in a lifetime, but do not smoke now) or “current smoking” (smoked more than 100 cigarettes in a lifetime and still smokes). Alcohol consumption was categorized as “non-drinkers” (consuming less than 12 drinks in a lifetime) or “drinkers” (consuming more than 12 drinks in a lifetime). Body mass index (BMI) was calculated by dividing body weight in kilograms by height squared in meters. It was then categorized as follows: normal weight (BMI < 25 kg/m²), overweight (25 ≤ BMI ≤ 30 kg/m²), or obesity (BMI > 30 kg/m²). Race was categorized as “white” (Mexican American, Other Hispanic, and Non-Hispanic White) or “other”. Household income to poverty line ratio (PIR) was categorized as low (PIR < 1), medium (1 ≤ PIR < 3), or high (PIR ≥ 3).

Diabetes mellitus was defined as a self-reported diagnosis with fasting blood glucose (FBG) ≥ 126 mg/dl or HbA1c level ≥6.5%, with insulin or oral hypoglycemic medications, according to the American Diabetes Association (ADA) diagnostic criteria (24). Hypertension was defined as a blood pressure ≥140/90 mmHg, a positive history of hypertension, or use of antihypertensive medications (25). The definitions of various medical conditions are based on specific criteria derived from a questionnaire regarding individuals' health histories. The definition of heart failure is articulated via the statement, “Someone has informed you that you have congestive heart failure”. The definition of angina pectoris is established via the statement, “Someone has informed you that you have angina pectoris”. The definition of coronary heart disease is derived from the statement, “Someone has informed you that you have coronary heart disease”. The definition of stroke is based on the statement, “Someone has informed you that you have experienced a stroke”. In addition, the questionnaire addresses various respiratory and musculoskeletal conditions. The definition of asthma is articulated via the statement, “Someone has informed you that you have asthma”. The definition of arthritis is based on the statement, “Someone has informed you that you have arthritis”. The definition of emphysema is established via the statement, “Someone has informed you that you have emphysema”. The definition of liver disease is derived from the statement, “Someone has informed you that you have any form of liver condition”. The definition of cancer is articulated via the statement, “Someone has informed you that you have cancer or a malignancy of any kind”. Finally, the definition of chronic kidney disease is based on the statement, “Someone has informed you that you have weak or failing kidneys”, or by an estimated creatinine clearance (eGFR) of less than 60 ml/min/1.73 m² (26). The WWI (cm/√kg) was calculated as waist circumference (cm) divided by the square root of weight (kg) (27).

### Statistical analysis

The measured variables of the study were presented as either mean (standard deviation) or median (quartiles) for continuous variables, and as frequency (percentage) for count variables. The normal distribution of variables was assessed using the Kolmogorov-Smirnov test. For normally distributed variables, the *t*-test or ANOVA was used to compare groups, while the Mann-Whitney *U*-test or Kruskal-Wallis *H*-test was used for non-normally distributed variables. The comparison of count variables was done using the chi-square test or Fisher's exact test. Weight-adjusted waist circumference index (WWI) was categorized into four quartiles (Q1, Q2, Q3, Q4), with the first quartile (Q1) designated as the reference quartile. The study utilized multivariate logistic regression analysis to assess the association between weight-adjusted waist circumference index (WWI) and the risk of myocardial infarction, with results expressed as odds ratio (OR) and 95% confidence interval (CI). The selection of covariates was based on factors associated with the risk of myocardial infarction in the previous literature, as well as potential confounders that may influence the relationship between weight-adjusted waist circumference index (WWI) and myocardial infarction. We considered the following covariates: gender, age, race, smoking, total smoking time, drinking, education, marital status, body mass index, ratio of family income to poverty, white blood cell count, segmented neutrophils percentage, red blood cells, hemoglobin, red cell distribution width, mean corpuscular volume, mean platelet volume, waist circumference, platelet count, total protein, albumin, alanine aminotransferase, aspartate aminotransferase, alkaline phosphatase, gamma-glutamyl transferase, blood urea nitrogen, creatinine, uric acid, glucose, triglycerides, lactate dehydrogenase, diabetes or prediabetes, hypertension, heart failure, coronary heart disease, angina pectoris, stroke, emphysema, liver disease, arthritis, asthma, chronic kidney disease, and cancer. In preliminary analyses, we first used a crude model to assess the association between WWI and myocardial infarction. Subsequently, we performed a minimally adjusted model, which adjusted for age and sex. Finally, we used a fully adjusted model, which included all the above covariates. A restricted cubic spline model was employed to evaluate possible nonlinear effects. Statistical significance was considered at *P* < 0.05. The data analysis was performed using R software (version 4.0.2, R Foundation) and Empower Stats (X&Y Solutions Inc.).

## Results

### Baseline characteristics

The prevalence of myocardial infarction was 4.82%. In comparison with the group without myocardial infarction, the myocardial infarction group exhibited lower levels of albumin, total cholesterol, total protein, erythrocyte count, hemoglobin, platelet count, and alcohol consumption. Additionally, they had higher levels of alkaline phosphatase, blood urea nitrogen, gamma-glutamyl transpeptidase, blood glucose, lactate dehydrogenase, triglycerides, uric acid, creatinine, white blood cell count, segmented neutrophils percent, mean erythrocyte volume, erythrocyte distribution width, mean platelet volume, weight-adjusted waist circumference index, age, smoking prevalence, and waist circumference. Furthermore, the myocardial infarction group had higher rates of diabetes, hypertension, coronary artery disease, heart failure, angina pectoris, stroke, asthma, arthritis, emphysema, liver disease, cancer, chronic kidney disease, and overweight (Table 1). Table 2 shows the baseline characteristics of participants in different WWI subgroups. Differences were found between the different quartiles of the WWI groups for most variables, except mean erythrocyte volume, mean platelet volume, and mean annual alcohol consumption.

**Table 1:** Baseline characteristics of participants reported myocardial infarction vs. those without reported myocardial infarction.

Variables	Reported myocardial infarction (*N* = 1,449)	Without reported myocardial infarction (*N* = 30,086)	*P-*value
Albumin (g/L)	41.56 (3.42)	42.66 (3.28)	<0.001
Alanine aminotransferase (U/L)	21.00 (17.00–27.00)	21.00 (17.00–29.00)	0.034
Aspartate aminotransferase (U/L)	23.00 (20.00–28.00)	23.00 (20.00–28.00)	0.020
Alkaline phosphatase (IU/L)	78.68 (28.04)	71.72 (25.66)	<0.001
Blood urea nitrogen (mmol/L)	5.71 (4.28–7.50)	4.60 (3.57–5.71)	<0.001
Total cholesterol (mmol/L)	4.88 (1.20)	5.16 (1.06)	<0.001
Gamma glutamyl transferase (U/L)	24.00 (17.00–37.00)	21.00 (15.00–32.00)	<0.001
Glucose (mmol/L)	6.30 (2.53)	5.51 (1.96)	<0.001
Lactate dehydrogenase (U/L)	142.62 (32.93)	134.09 (32.27)	<0.001
Total bilirubin (μmol/L)	12.83 (5.12)	12.56 (5.15)	0.054
Total protein (g/L)	71.96 (5.28)	72.80 (4.85)	<0.001
Triglycerides (mmol/L)	1.58 (1.06–2.35)	1.31 (0.88–1.99)	<0.001
Uric acid (μmol/L)	366.57 (98.62)	322.62 (83.83)	<0.001
Creatinine (μmol/L)	88.40 (71.60–106.08)	73.37 (61.88–88.40)	<0.001
WBC (×10^9^/L)	7.45 (2.30)	7.16 (2.37)	<0.001
Segmented neutrophils percent (%)	60.75 (9.77)	58.09 (9.41)	<0.001
Red cell count (×10^12^/L)	4.61 (0.54)	4.71 (0.50)	<0.001
Hemoglobin concentration (mg/dl)	14.21 (1.63)	14.34 (1.50)	0.002
Mean cell volume (fL)	91.32 (5.72)	89.80 (5.59)	<0.001
RDW (%)	13.39 (1.51)	12.81 (1.19)	<0.001
Platelet count (×10^9^/L)	244.07 (76.58)	263.93 (68.61)	<0.001
Mean platelet volume (fL)	8.16 (0.94)	8.11 (0.90)	0.017
WWI (cm/√kg)	11.49 (0.71)	10.95 (0.84)	<0.001
Age (years)	67.90 (12.64)	49.35 (18.12)	<0.001
Male (%)	973 (67.15%)	14,839 (49.32%)	<0.001
Race (%)			<0.001
Mexican American	171 (11.80%)	6,323 (21.02%)	
Other Hispanic	46 (3.17%)	1,801 (5.99%)	
Non-Hispanic White	970 (66.94%)	15,040 (49.99%)	
Non-Hispanic Black	214 (14.77%)	5,737 (19.07%)	
Other, including multi-racial	48 (3.31%)	1,185 (3.94%)	
Education [*n* (%)]			
Less than 9th grade	314 (21.67%)	4,126 (13.71%)	<0.001
9–11th grade	266 (18.36%)	4,800 (15.95%)	
High school graduate	342 (23.60%)	7,190 (23.90%)	
Some college	337 (23.26%)	8,155 (27.11%)	
College graduate or above	185 (12.77%)	5,771 (19.18%)	
Missing	5 (0.35%)	44 (0.15%)	
Marital status [*n* (%)]			<0.001
Married	799 (55.14%)	16,300 (54.18%)	
Widowed	319 (22.02%)	2,599 (8.64%)	
Divorced	171 (11.80%)	2,873 (9.55%)	
Separated	31 (2.14%)	944 (3.14%)	
Never married	65 (4.49%)	4,977 (16.54%)	
Living with partner	44 (3.04%)	2,009 (6.68%)	
Missing	20 (1.38%)	384 (1.28%)	
Smoking status			<0.001
Current smoking	836 (57.69%)	13,354 (44.39%)	
Former smoking	353 (24.36%)	12,230 (40.65%)	
Never smoking	260 (17.94%)	4,502 (14.96%)	
Total smoking time (years)	38.99 (22.00–52.00)	24.00 (12.00–38.00)	<0.001
Drinkers [*n* (%)]	950 (65.56%)	19,937 (66.27%)	0.033
Alcohol consumption/12 months (drinks)	24 (4–108)	24 (6–104)	0.192
PIR [*n* (%)]			<0.001
0–0.99	263 (18.15%)	5,075 (16.87%)	
1.00–2.99	704 (48.59%)	11,744 (39.03%)	
≧3.00	366 (25.26%)	10,998 (36.56%)	
Missing	116 (8.01%)	2,269 (7.54%)	
Diabetes or prediabetes [*n* (%)]	435 (30.02%)	3,378 (11.23%)	<0.001
Hypertension [*n* (%)]	975 (67.29%)	9,551 (31.75%)	<0.001
Heart failure [*n* (%)]	437 (30.16%)	503 (1.67%)	<0.001
Angina pectoris [*n* (%)]	508 (35.06%)	564 (1.87%)	<0.001
Coronary heart disease [*n* (%)]	714 (49.28%)	661 (2.20%)	<0.001
Stroke [*n* (%)]	242 (16.70%)	837 (2.78%)	<0.001
Asthma [*n* (%)]	238 (16.43%)	3,516 (11.69%)	<0.001
Arthritis [*n* (%)]	803 (55.42%)	7,714 (25.64%)	<0.001
Emphysema [*n* (%)]	145 (10.01%)	525 (1.74%)	<0.001
Liver disease [*n* (%)]	106 (7.32%)	970 (3.22%)	<0.001
Cancer [*n* (%)]	318 (21.95%)	2,528 (8.40%)	<0.001
Chronic kidney disease [*n* (%)]	513 (35.40%)	3,073 (10.21%)	<0.001
Waist circumference (cm)	103.59 (14.46)	97.63 (15.39)	<0.001
Body mass index (kg/m^2^)			<0.001
<25	340 (23.46%)	9,348 (31.07%)	
25.0–29.9	531 (36.65%)	10,643 (35.38%)	
30.0–34.9	349 (24.09%)	5,915 (19.66%)	
35	201 (13.87)	4,064 (13.51%)	
Missing	28 (1.93%)	116 (0.39%)	

WBC, white blood cell count; RDW, red cell distribution width; WWI, weight-adjusted waist circumference index; PIR, ratio of family income to poverty.

**Table 2:** Baseline characteristics of participants according to the WWI group.

Variables	WWI (Q1) (*N* = 7,871)	WWI (Q2) (*N* = 7,865)	WWI (Q3) (*n* = 7,866)	WWI (Q4) (*n* = 7,933)	*P*-value
Albumin (g/L)	43.75 (3.28)	42.97 (3.17)	42.28 (3.12)	41.44 (3.17)	<0.001
Alanine aminotransferase (U/L)	20.00 (16.00–27.00)	22.00 (17.00–30.00)	22.00 (17.00–30.00)	21.00 (17.00–28.00)	<0.001
Aspartate aminotransferase (U/L)	23.00 (19.00–27.00)	23.00 (20.00–28.00)	23.00 (20.00–28.00)	23.00 (20.00–27.00)	<0.001
Alkaline phosphatase (IU/L)	65.02 (21.42)	70.75 (25.05)	74.15 (26.31)	78.20 (28.17)	<0.001
Blood urea nitrogen (mmol/L)	4.40 (1.54)	4.62 (1.72)	4.97 (2.18)	5.45 (2.65)	<0.001
Total cholesterol (mmol/L)	4.88 (0.99)	5.21 (1.03)	5.24 (1.08)	5.25 (1.11)	<0.001
Gamma glutamyl transferase (U/L)	18.00 (13.00–26.00)	21.00 (15.00–33.00)	23.00 (16.00–36.00)	23.00 (16.00–35.00)	<0.001
Glucose (mmol/L)	4.98 (1.19)	5.32 (1.67)	5.70 (2.06)	6.19 (2.60)	<0.001
Lactate dehydrogenase (U/L)	129.30 (37.56)	133.04 (29.31)	136.26 (29.52)	139.30 (31.47)	<0.001
Total bilirubin (μmol/L)	13.34 (6.00) 11	12.65 (4.96)	12.41 (4.90)	11.91 (4.53)	<0.001
Total protein (g/L)	73.18 (4.81)	72.81 (4.84)	72.73 (4.80)	72.33 (5.00)	<0.001
Triglycerides (mmol/L)	0.95 (0.69–1.41)	1.28 (0.87–1.95)	1.47 (1.02–2.19)	1.65 (1.14–2.35)	<0.001
Uric acid (μmol/L)	303.85 (79.84)	322.73 (83.51)	332.05 (85.59)	339.79 (86.87)	<0.001
Creatinine (μmol/L)	79.56 (63.65–88.40)	74.26 (61.90–88.40)	72.49 (61.88–88.40)	72.49 (61.88–88.40)	<0.001
WBC (×10^9^/L)	6.66 (2.06)	7.09 (2.13)	7.35 (2.50)	7.59 (2.61)	<0.001
Segmented neutrophils percent (%)	56.74 (9.75)	57.90 (9.17)	58.54 (9.40)	59.66 (9.21)	<0.001
Red cell count (×10^12^/L)	4.75 (0.52)	4.75 (0.50)	4.71 (0.50)	4.63 (0.49)	<0.001
Hemoglobin concentration (mg/dl)	14.42 (1.48)	14.48 (1.50)	14.32 (1.52)	14.10 (1.51)	<0.001
Mean cell volume (fL)	89.85 (5.52)	89.90 (5.40)	89.77 (5.64)	89.96 (5.86)	0.164
RDW (%)	12.61 (1.15)	12.71 (1.08)	12.89 (1.23)	13.11 (1.34)	<0.001
Platelet count (×10^9^/L)	257.91 (62.50)	263.83 (67.32)	264.62 (70.35)	265.69 (75.38)	<0.001
Mean platelet volume (fL)	8.10 (0.89)	8.09 (0.87)	8.13 (0.90)	8.11 (0.93)	0.076
WWI (cm/√kg)	9.91 (0.38)	10.70 (0.16)	11.24 (0.17)	12.06 (0.42)	<0.001
Age (years)	38.05 (14.53)	46.69 (16.21)	53.72 (16.86)	62.25 (16.32)	<0.001
Male (%)	4,353 (55.30%)	4,324 (54.98%)	4,006 (50.93%)	3,129 (39.44%)	<0.001
Race (%)					<0.001
Mexican American	943 (11.98%)	1,611 (20.48%)	1,922 (24.43%)	2,018 (25.44%)	
Other Hispanic	377 (4.79%)	457 (5.81%)	485 (6.17%)	528 (6.66%)	
Non-Hispanic White	3,999 (50.81%)	4,008 (50.96%)	3,847 (48.91%)	4,156 (52.39%)	
Non-Hispanic Black	2,217 (28.17%)	1,431 (18.19%)	1,320 (16.78%)	983 (12.39%)	
Other, including multi-racial	335 (4.26%)	358 (4.55%)	292 (3.71%)	248 (3.13%)	
Education [n (%)]					<0.001
Less than 9th grade	420 (5.34%)	818 (10.40%)	1,220 (15.51%)	1,982 (24.99%)	
9–11th grade	1,087 (13.81%)	1,197 (15.22%)	1,369 (17.40%)	1,413 (17.82%)	
High school graduate	1,771 (22.50%)	1,938 (24.64%)	1,893 (24.07%)	1,930 (24.33%)	
Some college	2,536 (32.22%)	2,224 (28.28%)	2,068 (26.29%)	1,664 (20.98%)	
College graduate or above	2,051 (26.06%)	1,673 (21.27%)	1,308 (16.63%)	924 (11.65%)	
Missing	6 (0.08%)	15 (0.19%)	8 (0.10%)	18 (0.23%)	
Marital status [*n* (%)]					<0.001
Married	3,593 (46.37%)	4,559 (58.67%)	4,781 (61.47%)	4,166 (53.10%)	
Widowed	207 (2.67%)	384 (4.94%)	721 (9.27%)	1,606 (20.47%)	
Divorced	630 (8.13%)	811 (10.44%)	745 (9.58%)	858 (10.94%)	
Separated	242 (3.12%)	241 (3.10%)	273 (3.51%)	219 (2.79%)	
Never married	2,318 (29.92%)	1,203 (15.48%)	832 (10.70%)	689 (8.78%)	
Living with partner	756 (9.76%)	568 (7.31%)	422 (5.43%)	307 (3.91%)	
Missing	2 (0.03%)	4 (0.05%)	4 (0.05%)	1 (0.01%)	
Smoking status					<0.001
Current smoking	3,401 (43.21%)	3,560 (45.26%)	3,577 (45.47%)	3,652 (46.04%)	
Former smoking	3,383 (42.98%)	3,136 (39.87%)	3,049 (38.76%)	3,015 (38.01%)	
Never smoking	1,087 (13.81%)	1,169 (14.86%)	1,240 (15.76%)	1,266 (15.96%)	
Total smoking time (years)	17.00 (15.00–19.00)	17.00 (15.00–19.00)	17.00 (15.00–20.00)	17.00 (15.00–20.00)	<0.001
Drinkers [*n* (%)]	5,715 (77.30%)	5,436 (73.57%)	5,124 (69.02%)	4,612 (61.23%)	
Alcohol consumption/12 months (drinks)	2.00 (1.00–3.00)	2.00 (1.00–3.00)	2.00 (1.00–3.00)	2.00 (1.00–3.00)	0.245
PIR [*n* (%)]					<0.001
0–0.99	1,172 (14.89%)	1,238 (15.74%)	1,270 (16.15%)	1,658 (20.90%)	
1.00–2.99	2,667 (33.88%)	2,929 (37.24%)	3,240 (41.19%)	3,612 (45.53%)	
≧3.00	3,474 (44.14%)	3,125 (39.73%)	2,759 (35.08%)	2,006 (25.29%)	
Missing	558 (7.09%)	573 (7.29%)	597 (7.59%)	657 (8.28%)	
Diabetes or prediabetes [*n* (%)]	236 (3.00%)	545 (6.93%)	1,062 (13.50%)	1,970 (24.83%)	<0.001
Hypertension [*n* (%)]	1,174 (14.92%)	2,059 (26.18%)	3,042 (38.67%)	4,251 (53.59%)	<0.001
Heart failure [*n* (%)]	54 (0.69%)	142 (1.81%)	275 (3.50%)	469 (5.91%)	<0.001
Angina pectoris [*n* (%)]	66 (0.84%)	212 (2.70%)	296 (3.76%)	498 (6.28%)	<0.001
Coronary heart disease [*n* (%)]	80 (1.02%)	251 (3.19%)	450 (5.72%)	498 (6.28%)	<0.001
Stroke [*n* (%)]	83 (1.05%)	168 (2.14%)	299 (3.80%)	529 (6.67%)	<0.001
Asthma [*n* (%)]	976 (12.40%)	850 (10.81%)	854 (10.86%)	1,074 (13.54%)	<0.001
Arthritis [*n* (%)]	968 (12.30%)	1,708 (21.72%)	2,393 (30.42%)	3,448 (43.46%)	<0.001
Emphysema [*n* (%)]	48 (0.61%)	110 (1.40%)	175 (2.22%)	337 (4.25%)	<0.001
Liver disease [*n* (%)]	186 (2.36%)	245 (3.12%)	305 (3.88%)	340 (4.29%)	<0.001
Cancer [n (%)]	365 (4.64%)	558 (7.09%)	839 (10.67%)	1,084 (13.66%)	<0.001
Chronic kidney disease [n (%)]	313 (3.98%)	577 (7.34%)	977 (12.42%)	1,719 (21.67%)	<0.001
Waist circumference (cm)	84.45 (10.21)	94.69 (10.98)	102.07 (12.24)	110.30 (14.50)	<0.001
Body mass index (kg/m^2^)					<0.001
<25	4,598 (58.47%)	2,578 (32.82%)	1,558 (19.89%)	941 (12.01%)	
25.0–29.9	2,370 (30.14%)	3,213 (40.90%)	3,046 (38.88%)	2,535 (32.35%)	
30.0–34.9	666 (8.47%)	1,466 (18.66%)	1,961 (25.03%)	2,186 (27.89%)	
35	230 (2.92%)	598 (7.61%)	1,270 (16.21%)	2,175 (27.75%)	
Missing	7 (0.09%)	10 (0.13%)	31 (0.39%)	96 (1.21%)	

WBC, white blood cell count; RDW, red cell distribution width; WWI, weight-adjusted waist circumference index; PIR, ratio of family income to poverty.

### Association between the WWI and myocardial infarction

**Figure 2:**
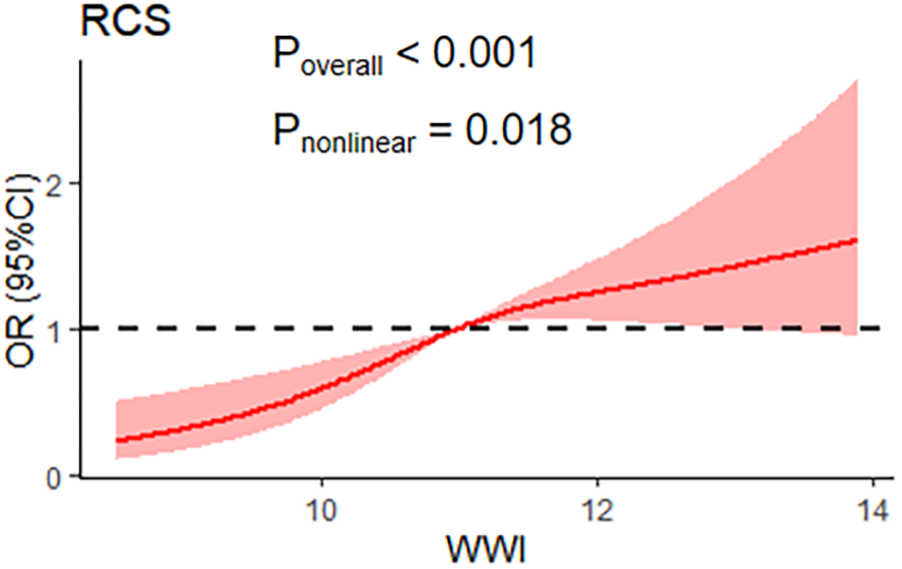
Restricted cubic spline for the association between the weight-adjusted waist circumference index (WWI) and myocardial infarction.

Logistic regression analysis was conducted to evaluate the predictive impact of WWI on the prevalence of myocardial infarction, and the findings are presented in Table 3. In the unadjusted model, the risk of myocardial infarction increased by 2.14-fold for every 1-cm/√kg increase in WWI. Following adjustment for age and gender, the risk decreased to 1.39-fold for each 1-cm/√kg increase in WWI, and further decreased to 1.34-fold when other covariates such as smoking status, serum albumin, alanine aminotransferase, and aspartate aminotransferase were included. When WWI was categorized into quartiles, the risk of myocardial infarction exhibited a significantly increasing trend with rising quartiles of WWI (*P* for trend = 0.003), and the risk of myocardial infarction was 1.81-fold higher in the fourth quartile compared to the first quartile. In sensitivity analyses excluding the obese population, we included 20,839 non-obese participants. Of these participants, 871 reported myocardial infarction. The fully adjusted model showed that the association between WWI and myocardial infarction remained significant, with an OR of 1.40 (95% CI: 1.13–1.74), *P* < 0.05. This suggests that higher WWI is associated with an increased risk of myocardial infarction, even in the non-obese population. Additionally, restricted cubic spline analysis revealed a nonlinear positive correlation between WWI and myocardial infarction (*P* = 0.003) (Figure 2). A significant threshold effect was observed: when WWI was less than 10.97 cm/√kg, the risk of myocardial infarction increased by 2.29-fold (OR = 2.29, 95% CI: 1.37–3.84); when WWI was higher than 10.97 cm/√kg, the upward trend in the risk of myocardial infarction significantly slowed with increasing WWI (OR = 1.26, 95% CI: 1.03–1.56). The optimal WWI threshold for predicting myocardial infarction was >11.15 cm/√kg [area under the curve (AUC) = 0.690, sensitivity 69.43%, specificity 59.96%, *P* < 0.001]. In comparison, the optimal BMI threshold for predicting myocardial infarction was >26.16 kg/m² (AUC = 0.541, sensitivity 68.54%, specificity 39.71%, *P* < 0.001), and the best cut-off value for waist circumference to predict myocardial infarction was >104.4 cm (AUC = 0.620, sensitivity 58.45%, specificity 60.53%, *P* < 0.001). Additionally, the area under the receiver-operating characteristic (ROC) curve for WWI was significantly higher than that for waist circumference (*P* = 0.003) and BMI (*P* = 0.007).

**Table 3:** Association between WWI and the reported myocardial infarction.

Variable	Crude model (OR; 95% CI; *P-*value)	Minimally adjusted model (OR; 95% CI; *P-*value)	Fully adjusted model 1 (OR; 95% CI; *P-*value)	Fully adjusted model 2^a^ (OR; 95% CI; *P-*value)
WWI	2.14 (2.01, 2.28) <0.001	1.39 (1.28, 1.50) <0.001	1.34 (1.13, 1.58) <0.001	1.40 (1.13, 1.74) 0.002
WWI(quartile)				
Q1 (7.90–10.39)	Reference	Reference	Reference	Reference
Q2 (10.40–10.96)	2.78 (2.18, 3.55) <0.001	1.59 (1.24, 2.05) <0.001	1.38 (1.01, 1.87) 0.040	1.25 (0.89, 1.77) 0.199
Q3 (10.97–11.54)	5.11 (4.06, 6.43) <0.001	2.03 (1.60, 2.58) <0.001	1.59 (1.15, 2.20) 0.005	1.43 (0.97, 2.09) 0.069
Q4 (11.55–15.52)	8.04 (6.44, 10.04) <0.001	2.36 (1.86, 3.00) <0.001	1.81 (1.24, 2.63) 0.002	1.70 (1.08, 2.69) 0.022
*P* for Trend	<0.001	<0.001	0.003	0.020

OR, odds ratio; CI, confidence interval.

The crude model was unadjusted.

The minimally adjusted model was adjusted for gender and age.

The fully adjusted model 1 was adjusted for gender, age, race, smoking, total smoking time, drinking, education, marital status, body mass index, ratio of family income to poverty, white blood cell count, segmented neutrophils percent (%), red blood cell, hemoglobin, red cell distribution width, mean cell volume, mean platelet volume, waist circumference, platelet count, total protein, albumin, alanine aminotransferase, aspartate aminotransferase, alkaline phosphatase, gamma glutamyl transferase, blood urea nitrogen, creatinine, uric acid, glucose, triglycerides, lactate dehydrogenase, diabetes or prediabetes, hypertension, heart failure, coronary heart disease, angina pectoris, stroke, emphysema, liver disease, arthritis, asthma, chronic kidney disease, and cancer.

^a^ Fully adjusted model 2 excludes the obese population and includes the same adjustment variables as fully adjusted model 1.

### Subgroup analysis

**Figure 3:**
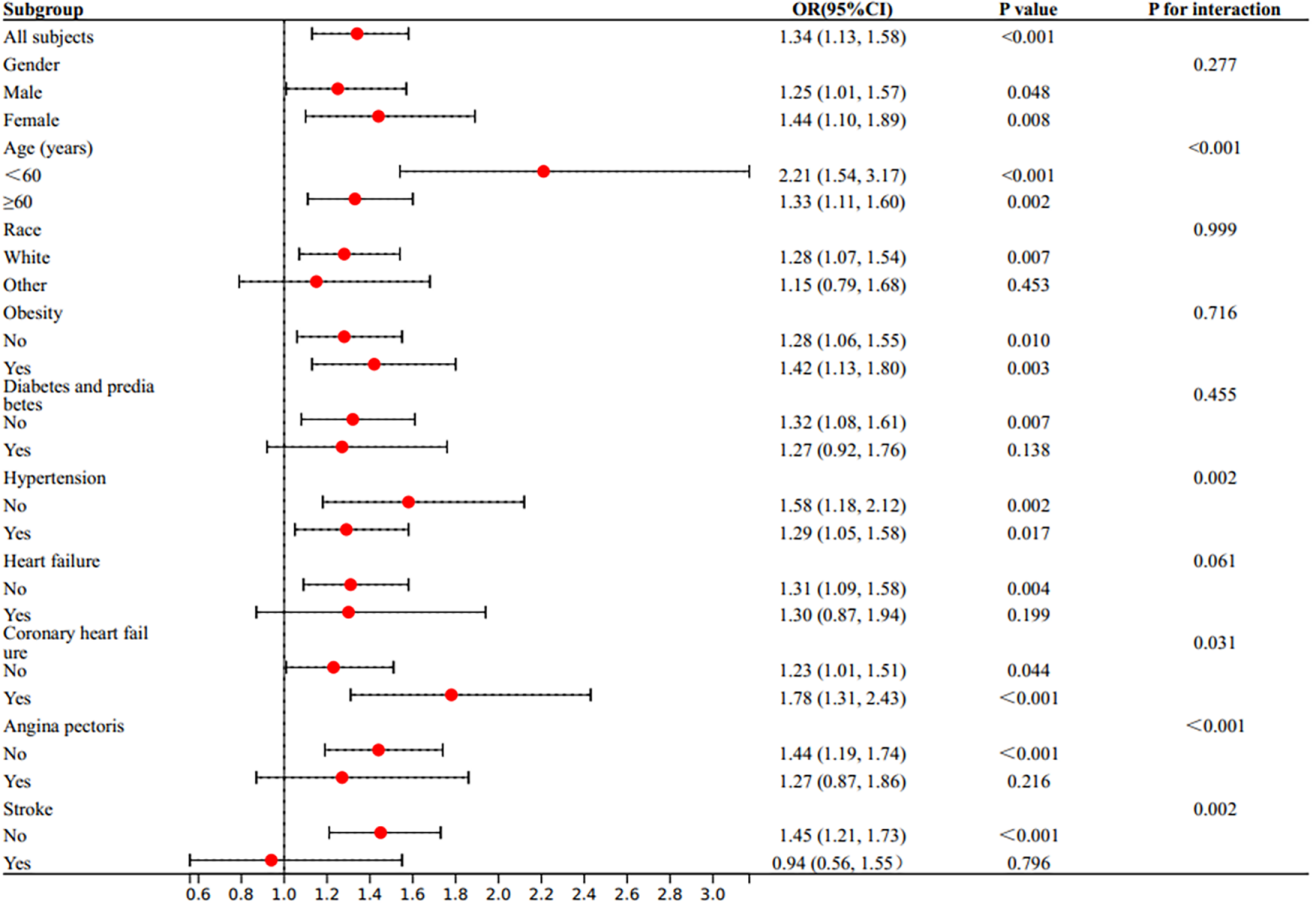
Subgroup analysis for the association between weight-adjusted waist circumference index (WWI) and the prevalent reported myocardial infarction. The multivariate logistic model adjusted for all variates in used in the fully adjusted model of Table 2, except for the variate that used to define subgroups.

Subgroup analyses and interaction tests were conducted to evaluate the association between WWI and myocardial infarction across various subgroups, including gender, age, race, obesity, diabetes mellitus, hypertension, heart failure, coronary heart disease, angina pectoris, and stroke, to assess the robustness of the association and potential population differences (Figure 3). The impact of WWI on myocardial infarction exhibited variations across different populations, with the effect being different for age groups (<60 years: OR = 2.21, 95% CI: 1.54–3.17; age ≥60 years: OR = 1.33, 95% CI: 1.11–1.60), hypertension (yes: OR = 1.29, 95% CI: 1.05–1.58; no: OR = 1.58, 95% CI: 1.18–2.12), coronary heart disease (yes: OR = 1.78, 95% CI: 1.31–2.43; no: OR = 1.23, 95% CI: 1.01–1.51), angina pectoris (yes: OR = 1.27, 95% CI: 0.87–1.86; no: OR = 1.44, 95% CI: 1.19–1.74), and stroke (yes: OR = 0.94, 95% CI: 0.56–1.55; no: OR = 1.45, 95% CI: 1.21–1.73). The test results indicated an interaction between the incidence of myocardial infarction and WWI associated with age, hypertension, coronary heart disease, angina pectoris, and stroke (*P* for interactio*n* <0.05), while no interaction was observed with gender, race, obesity, diabetes or prediabetes, and heart failure (*P* for interaction >0.05).

## Discussion

Myocardial infarction is a severe cardiovascular disease that is closely linked to obesity and other factors. The World Health Organisation (WHO) diagnoses obesity based on a body mass index (BMI) over 30 kg/m². However, BMI has limitations in distinguishing between fat mass and lean body mass (28). New indicators of obesity, such as the body shape index (ABSI), waist-to-hip ratio, and waist-to-height ratio, have been successively reported. The ABSI is associated with cardiovascular risk (29), and the waist-to-hip ratio is positively associated with subclinical myocardial injury (30). Waist-to-hip and waist-to-height ratios outperform BMI in predicting cardiovascular disease (CVD) (11). Waist-to-hip ratio and waist-to-height ratio may mask the effect of weight on health, whereas the weight-adjusted waist index (WWI) not only measures waist circumference but also takes into account an individual's weight. This makes WWI more comprehensive and accurate in assessing obesity levels.

The study findings indicate a significant correlation between the WWI and the risk of myocardial infarction (MI) in a representative US population. The possible mechanisms are that WWI, as an indicator of central obesity, is associated with a pro-inflammatory state and endothelial dysfunction. In obesity, dysfunctional adipose tissue releases pro-inflammatory adipocytokines such as TNFα, IL-6, IL-1β, and free fatty acids (FFAs), which increase levels of pro-inflammatory factors (31). The increase in these pro-inflammatory factors triggers an inflammatory response, which in turn leads to endothelial dysfunction. Endothelial dysfunction increases lipoprotein permeability, which leads to subendothelial accumulation of lipoproteins, leukocyte recruitment, and platelet activation, ultimately triggering atherosclerosis and increasing the risk of myocardial infarction (32). In addition, obesity induces systemic oxidative stress through a variety of biochemical pathways, and oxidative stress can damage cardiomyocytes (33), further increasing the risk of myocardial infarction. After adjusting for confounding factors, such as demographic characteristics, physical measurements, laboratory indices, and medical history, multivariate logistic regression analyses revealed a significant positive association between WWI and the risk of myocardial infarction, even after controlling for variables such as BMI and waist circumference. Specifically, for every 1 cm/√kg increase in WWI, the risk of myocardial infarction increased by 34%. The positive correlation remained stable when WWI was further divided into quartiles. This finding is consistent with the association of WWI with the risk of heart failure (34) and stroke (35). Our sensitivity analyses excluded the obese population to assess whether the association between WWI and myocardial infarction was influenced by obesity status. In the nonobese population, we found that the positive association between WWI and myocardial infarction remained, suggesting that WWI may be a risk factor for myocardial infarction independent of obesity. Therefore, the association between WWI and the risk of myocardial infarction was independent of the confounding effects of total and abdominal obesity, highlighting the influence of fat accumulation on the risk of myocardial infarction. The relationship between WWI and the incidence of myocardial infarction was found to be nonlinear, similar to the results of studies related to chronic kidney disease (36). When WWI was ≥10.97 cm/√kg, it was associated with a higher incidence of myocardial infarction, similar to the thresholds reported in studies of hypertension (37). When WWI was below 10.97 cm/√kg, an increase in WWI was associated with a higher risk of myocardial infarction. This correlation was attenuated when WWI was above this threshold. The efficacy of WWI in predicting myocardial infarction was superior to that of BMI and waist circumference, as WWI more accurately reflects central obesity caused by visceral adipose deposits. Visceral adipocytes release unknown factors that may promote systemic atherosclerosis and increase the risk of myocardial infarction (38). The optimal threshold for WWI to predict myocardial infarction was slightly higher at 11.15 cm/√ kg, compared to the threshold for predicting unhealthy body composition (39).

Subgroup analyses revealed that an increase in WWI was significantly associated with a higher risk of myocardial infarction in women, while this association was not significant in men. However, further research is needed to confirm these findings. This finding is consistent with previous reports in the literature (40), which suggest that female subjects may be more susceptible to increases in WWI and latent adiposity. The study found that WWI was an independent risk factor for myocardial infarction across all age groups, with a particularly significant impact on those under 60 years old. This suggests that abdominal fat accumulation may pose a greater risk to younger individuals, while the effect of WWI on myocardial infarction decreases with age.

Furthermore, the relationship between WWI and the risk of myocardial infarction varied among different disease types. Although the OR for the association of WWI with myocardial infarction was higher in whites than in nonwhites, there was no interaction between race and WWI, suggesting that race did not significantly influence the association of WWI with myocardial infarction. In subgroups of different sexes, obesity, hyperglycemia, and heart failure, characteristics were also demonstrated, i.e., these subgroups did not interact with WWI. However, in hypertension, angina, coronary artery disease, and stroke, there was an interaction with WWI, suggesting that we should take into account the potential impact of the patient's disease state when assessing the relationship between WWI and myocardial infarction.

This study has several strengths. The use of NHANES, a high-quality data source with rigorous methodology, is a major strength of this study. Secondly, the study employed multivariate statistical analyses to comprehensively explore the linear, nonlinear, and dose-response relationships between WWI and the risk of myocardial infarction, thus achieving a more comprehensive understanding of this relationship. Finally, the study was able to more accurately determine the independent relationship between WWI and myocardial infarction by comprehensively adjusting for potential confounders such as socioeconomic status, education level, race, comorbidities, and laboratory parameters, thereby strengthening the robustness of the findings.

Despite the strengths of this study, it is important to recognize its limitations. This study relied on retrospective data and may be subject to information bias. In addition, our sample primarily consisted of individuals from the United States, which may limit the generalizability of the results. Participants' self-reported data, such as disease status and lifestyle factors, may have introduced recall bias. Future studies should consider the use of prospective designs and more diverse and representative samples to enhance the generalizability and reliability of results.

## Conclusion

The weight-adjusted waist index (WWI), a new measure of obesity, has shown promise in predicting the risk of cardiovascular diseases, including myocardial infarction. We recommend incorporating WWI into routine physical examinations and cardiovascular risk screening as an early warning mechanism. This may facilitate early identification of high-risk individuals and promote earlier preventive interventions.

## Data Availability

The original contributions presented in the study are included in the article/Supplementary Material, further inquiries can be directed to the corresponding author.
